# High resolution measurement of DUF1220 domain copy number from whole genome sequence data

**DOI:** 10.1186/s12864-017-3976-z

**Published:** 2017-08-14

**Authors:** David P. Astling, Ilea E. Heft, Kenneth L. Jones, James M. Sikela

**Affiliations:** 1 Department of Biochemistry and Molecular Genetics, University of Colorado School of Medicine, Aurora, CO USA; 2 Department of Pediatrics, University of Colorado School of Medicine, Aurora, CO USA

**Keywords:** Copy number variation, CNV, DUF1220, Genome informatics, Next-generation sequencing, Bioinformatics

## Abstract

**Background:**

DUF1220 protein domains found primarily in Neuroblastoma BreakPoint Family (*NBPF*) genes show the greatest human lineage-specific increase in copy number of any coding region in the genome. There are 302 haploid copies of DUF1220 in hg38 (~160 of which are human-specific) and the majority of these can be divided into 6 different subtypes (referred to as clades). Copy number changes of specific DUF1220 clades have been associated in a dose-dependent manner with brain size variation (both evolutionarily and within the human population), cognitive aptitude, autism severity, and schizophrenia severity. However, no published methods can directly measure copies of DUF1220 with high accuracy and no method can distinguish between domains within a clade.

**Results:**

Here we describe a novel method for measuring copies of DUF1220 domains and the *NBPF* genes in which they are found from whole genome sequence data. We have characterized the effect that various sequencing and alignment parameters and strategies have on the accuracy and precision of the method and defined the parameters that lead to optimal DUF1220 copy number measurement and resolution. We show that copy number estimates obtained using our read depth approach are highly correlated with those generated by ddPCR for three representative DUF1220 clades. By simulation, we demonstrate that our method provides sufficient resolution to analyze DUF1220 copy number variation at three levels: (1) DUF1220 clade copy number within individual genes and groups of genes (gene-specific clade groups) (2) genome wide DUF1220 clade copies and (3) gene copy number for DUF1220-encoding genes.

**Conclusions:**

To our knowledge, this is the first method to accurately measure copies of all six DUF1220 clades and the first method to provide gene specific resolution of these clades. This allows one to discriminate among the ~300 haploid human DUF1220 copies to an extent not possible with any other method. The result is a greatly enhanced capability to analyze the role that these sequences play in human variation and disease.

**Electronic supplementary material:**

The online version of this article (doi:10.1186/s12864-017-3976-z) contains supplementary material, which is available to authorized users.

## Background

Highly duplicated sequences, including genes, are prevalent throughout the human genome [1]. While they have been linked to important evolutionary [2, 3] and medical phenotypes [4], they often go unexamined in studies of genetic disease due to their complexity. Thus, there is a growing need to develop improved strategies for accurate copy number determination of highly duplicated sequences. While a number of methods exist for scoring copy number variations (CNVs) (e.g. array comparative genomic hybridization (arrayCGH), SNP arrays, qPCR, ddPCR and read depth from exome sequencing) these methods are not ideal for high-resolution measurement of DUF1220 domains due to limitations in throughput, accuracy and/or coverage. The primary challenge for both array based methods and exome sequencing lies in the hybridization efficiency of each probe with its respective target and thus causing variance and resulting uneven coverage, systematic bias, and inaccuracy of the measurement [5, 6]. More recently the use of whole genome sequencing (WGS) to estimate copy number by sequence read depth has become more prevalent with the increasing availability of WGS datasets. However, accurate copy number estimation for highly duplicated sequences remains a challenge [7] and, as a result, many CNV methods mask highly duplicated sequences and segmental duplications from the analysis [5, 8, 9]. Furthermore, previous reports have focused largely on measurement of gene copy number changes but sequences can vary as a result of both gene dosage changes and intragenic domain expansion/contraction. Consideration of this fact is important for two reasons. First, intragenic sequence gains or losses can confound estimates of gene copy number, and second, changes in copy number arising from intragenic changes may have different phenotypic effects than those arising from gene dosage changes.

Among the most interesting examples of highly duplicated human genome sequences are those encoding DUF1220 protein domains. Sequences encoding DUF1220 domains show the greatest human lineage-specific increase in copy number of any protein coding region in the genome [2]. The copy number of DUF1220 shows a dramatic increase specifically among anthropoid primates (monkeys, apes and humans), with the most extreme increase in copy number occurring in the human lineage (humans: ~300 haploid copies; great apes: 97–138; monkeys: 48–75; all other mammals: 1–9) [10, 11]. Our lab and others have previously shown that among primate species, an increase in copy number is associated, in a DNA dose-dependent manner, with an evolutionary increase in brain size, cortical neuron number and several other brain-related phenotypes [11–14].

The great majority of human DUF1220 domains are encoded by the *NBPF* gene family [15], map primarily to the 1q21 region in humans, and can be subdivided into six different subtypes, or clades, based on sequence similarity [10]. Three of the clades are conserved across primates (CON1–3) and three show intragenic copy number increases specific to the human lineage (HLS1–3). Interestingly the clades follow a generally fixed arrangement within each *NBPF* gene: From 5′ to 3′ (from N-terminus to C-terminus in the predicted protein) they almost always occur in the following order: CON1, CON2, HLS1, HLS2, HLS3, and CON3 [16]. In human populations, copies of DUF1220 sequences show a Gaussian distribution that represents a rich, and largely unexamined, source of functional allelic variation [17].

We believe that the measurement of clade-specific copy number is essential because increases in the copy number of specific DUF1220 clades have been correlated with several important cognitive phenotypes. These include gray matter volume in a non-disease human population (CON1 and CON2) [14], autism severity (CON1) [17, 18], schizophrenia severity (CON1 and HLS1) [19] and cognitive aptitude (CON2) [20]. These associations would be obscured if only total DUF1220 copy number or *NBPF* gene copy number was examined. Gene-specific resolution is also important, as knowledge of which DUF1220 domains are changed in copy number, where they are located, and how they changed (gene duplication/deletion or intragenic domain expansion/contraction) may be critical to identifying relationships between copy number and disease. In addition to measuring gene copy number and genomic DUF1220 clade copy number, this requires the measurement of DUF1220 domain copy number within each gene.

While other groups have measured CNVs from WGS data, measurement of the *NBPF* genes and DUF1220 domains has been limited and to our knowledge, none have reported on clade specific copy number of DUF1220 domains. We know of two studies that have reported *NBPF* gene copy number variation [21] and one reporting DUF1220 copy number variation [22]. These studies were limited in their scope and resolution of *NBPF* gene copy number. Sudmant et al. (2010) [21] reported on only 9 of the 24 *NBPF* genes, while Sudmant et al. (2015) [22] reported population stratification of *NBPF* gene copy number without specifying which *NBPF* genes were involved. Sudmant et al. (2013) [23] reported on DUF1220 copy number within *NBPF10* [22], however the values reported are consistent with haploid genome-wide DUF1220 copy number rather than *NBPF10* DUF1220 copy number.

At the basis of accurate quantification of read depth and copy number estimation lies the alignment strategy that is used to map reads back the genome reference. Previous studies have utilized a strategy which finds all possible alignments for each read has been used often [21, 24, 25]. In brief, this strategy tries to maximize read ambiguity and cross alignment between different duplicated segments by shortening longer reads to 36 bp single-end reads and finding all possible alignments within two mismatches. The strength of this method is that it provides an aggregate measure of highly duplicated sequences as was demonstrated by Sudmant et al. 2010 [21]. However, this method lacks specificity within highly homologous segmental duplications. This can be partially addressed by the use of Singly Unique Nucleotides (SUN) identifiers [21], as long as there are enough diagnostic SUN positions for each region. Due to their highly-duplicated nature, many DUF1220 domains lack single base differences so they would not be measured with a SUN-based approach. Another limitation of the strategy of finding all possible alignments is that it is seven times slower than finding the best alignment and the resulting alignment files are often two orders of magnitude larger. We set out to test the accuracy and resolution of DUF1220 copy number measurement that can be obtained with this method and explore the possibility of aligning longer reads with increased specificity. The specificity would allow for the quantification of individual domains and DUF1220 sequences within genes.

In this study, we explore how these strategies and various sequencing parameters affect the accuracy and precision of copy number estimation and demonstrate a method in which copies of DUF1220 and DUF1220 encoding genes can be accurately estimated. We validate this method with simulations, ddPCR, and apply it to data from the 1000 Genomes Project. We demonstrate not only the accurate estimation of DUF1220-clade specific copies, but also the delineation of clades, and in some cases domains, within individual *NBPF* genes. Such information allows one to determine if variations are due to changes in the copy number of whole genes or intragenic domain copy number expansions or contractions within specific individual *NBPF* genes. Together these advances allow us to utilize whole genome sequence data to identify copy number changes in DUF1220 sequences with unprecedented accuracy and precision, allowing potential disease associations to be examined at the highest level of resolution so far reported.

## Results

### Characterizing the read alignment ambiguity between DUF1220 domains

**Fig. 1:**
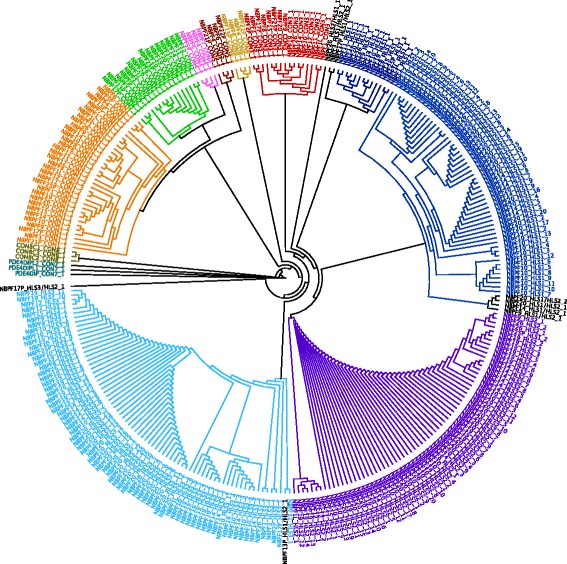
DUF1220 domains cluster by sequence similarity into six major clades. A Neighbor-Joining tree of DUF1220 domain protein sequences was constructed with Geneious v. 10.0.5. Branch colors represent the clade that each DUF1220 domain has been assigned to. DUF1220 domains for which the sequence is a hybrid of two major clades are in black. The aligned sequence data supporting the clade assignments can be found in Additional file 6

Because some of the ~300 DUF1220 copies in the haploid human genome display high sequence similarity to one another [10], it is likely that some sequence reads will map equally well to multiple locations. To further understand the relationship and sequence conservation between each of the 24 NBPF genes and respective domains or subtypes, we performed a detailed annotation of the *NBPF* genes in the most recent version of the human genome (hg38). We then used the sequences to carry out a detailed sequence analysis and clustering (Fig. 1). We have included the 8 predicted *NBPF* pseudogenes (as annotated in hg38) in our analysis as their domains may have impacts not related to their coding potential (e.g. substrates for homologous recombination, targets of DNA or RNA binding proteins). Application of the method described in this paper to future analysis of variation and disease associations allows one to measure the DUF1220 domains of pseudogenes separately from those that are predicted to be protein-encoding.

**Fig. 2:**
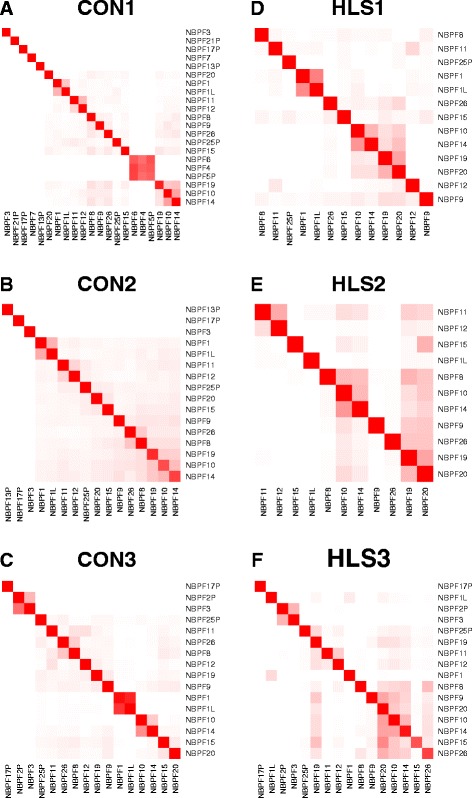
Read alignment ambiguity between *NBPF* genes using the ‘best’ alignment strategy with Bowtie2. For each clade (a-f), simulated reads from individual domains (*columns*) were mapped back to the reference genome and the percentage of reads aligning to each domain was calculated (*rows*). The *shade of red* is proportional to the percentage of reads aligning to each gene (*solid red* = 100% alignment and *white* = 0% alignment)

To develop our methodology for measuring DUF1220 copies, we wanted to determine the extent to which this read alignment ambiguity occurs. We carried out a simulation in which 100 bp paired-end reads from each DUF1220 domain were generated from the human reference genome, hg38, and aligned back to the reference to determine the extent to which reads from each domain (CON1, CON2, CON3, HLS1, HLS2, and HLS3) selectively align to the correct gene and clade. We found that, with 100 bp paired-end reads, the DUF1220 sequences from eight genes can be uniquely measured; 100% of the reads originating from them align to the originating gene and clade (e.g. *NBPF7*) (Fig. 2). In other cases, a proportion of the reads align equally well to two or more genes that have high sequence similarity (e.g. *NBPF4, NBPF5P & NBPF6)* (Fig. 2). Simulations involving 300 and 600 bp paired-end reads could not resolve the domains within *NBPF4*, *NBPF5P*, and *NBPF6*. If not accounted for, this read alignment ambiguity would result in over- or under- estimates of gene-specific clade copy number.

To address the challenge of read alignment ambiguity, we observed from our simulation that show read sharing is restricted to small clusters of genes and not distributed across all genes. By grouping related genes together for analysis, one can maintain accuracy and improve our resolution of copies within a clade. For example, we calculate the number of CON1 domains from *NBPF4, NBPF5,* and *NBPF6* together because they share a high percentage of their reads. Likewise, domains from *NBPF10, NBPF14, NBPF19, and NBPF20* share alignment ambiguity, so copies for these genes can be aggregated. As described below, this approach substantially reduces the error in copy number measurement. Additional file 1: Table S1 shows a strategy for grouping related genes into 60 categories based on the results from the simulated data for 100 bp paired end reads (Fig. 2). While grouping genes with high read sharing reduces resolution, the level of resolution obtainable with 100 bp paired-end reads is still an improvement over existing methods. The most appropriate gene groups to use for any given analysis will depend on the goals of the researcher (e.g. whether accuracy or resolution is a priority) and the sequence data available, as longer paired-end reads should improve the ability to localize reads to the correct gene (and vice versa for shorter sequencing reads).

### Establishment of four levels of DUF1220 measurement

Based on the read alignment ambiguity shown in Fig. 2, we differentiated four levels of resolution at which DUF1220 copy number can be measured; 1) Domain level measures are of each individual DUF1220 domain, i.e. alignment to a precise genomic location, 2) Gene-specific clade level measures are of all domains from a particular clade that occur within each DUF1220-encoding gene, i.e. reads align to a particular clade within an NBPF gene (Additional file 1: Table S1) 3) Group-specific measures are of all domains from a particular clade that occur within gene grouping as described above (Additional file 1: Table S1), 4) Clade-specific measures are all DUF1220 domains belonging to each of the 6 different DUF1220 clades.

### Evaluation of read length and paired-end reads on quantification of DUF1220 copies

**Fig. 3:**
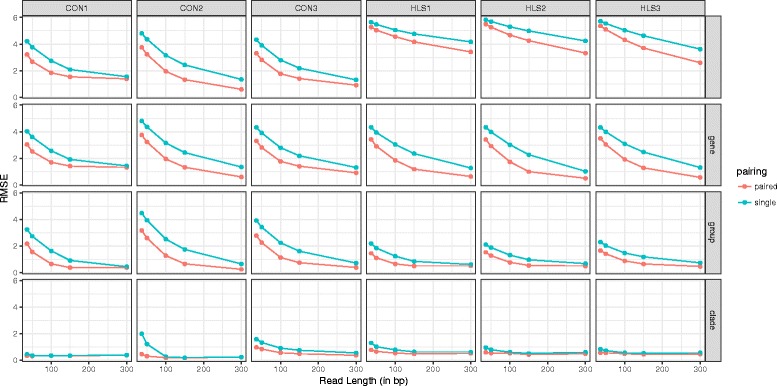
Effect of sequencing parameters on the accuracy and precision of the measurement. Simulated reads were generated for each domain and aligned back to the genome. RMSE was calculated at the domain level (do reads align to the exact genomic location from where they originated?), at the gene-specific clade level (do reads align to the correct clade within the originating *NBPF* gene?), at the group-specific clade level (to the correct clade within gene groupings described above), and at the clade-specific level (do reads align to the correct clade?)

**Fig. 4:**
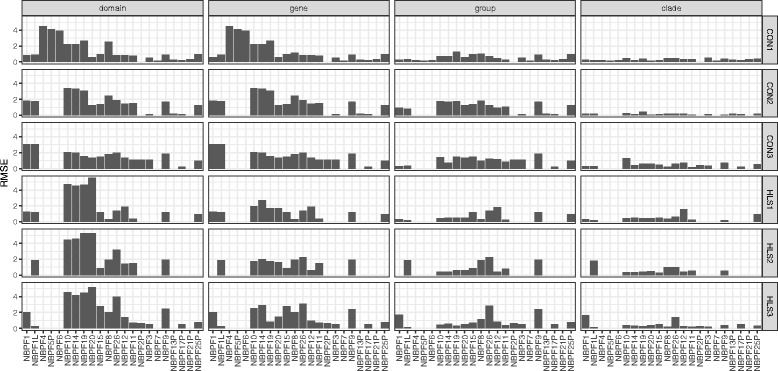
Variation in Root Mean Squared Error (RMSE) of the copy number estimate by gene and clade. Simulated data were generated for 100 bp paired end reads and the RMSE for each gene was calculated for each of four levels of resolution as described in the methods

In order to measure DUF1220 copies, we need to determine which kind of sequencing data would be most applicable and how sequencing parameters may influence the accuracy and precision of the measurement. Previous strategies have relied on very short 36 bp reads, we hypothesized that longer reads would improve accuracy of copy number prediction. To address this, we compared the effect of read length, as well as single and paired-end reads, on the accuracy of our read depth estimate based on simulated data. We simulated reads from the sequences of each of the DUF1220 domains based on the human reference genome hg38 and aligned these back to the genome along with additional levels of the reads spiked in. The simulated read lengths were 36, 100, 150 or 300 bp long, both single- and paired end. For each of the read lengths, we compared the predicted and measured coverage and report the combined root mean squared error (RMSE) of the prediction for each of the four different levels of resolution (Fig. 3). Fig. 4 shows the average RMSE for domains within each gene when 100 bp, paired-end reads are utilized. A potential limitation of calculating the RMSE for the spike-in study is that the variances may not scale linearly for domains where the off-target alignment rate is high. In some cases we observed that the absolute difference between the measured and simulated copy numbers to increase with increasing simulated coverage. By using the relative ratio between measured and simulated copy numbers the respective off-target alignments remain the same and are compared consistently throughout the entire simulation experiment (e.g. if for a particular domain, 10% of the reads align off target, one would measure a copy number of 0.9 for 1 simulated copy, and a copy number of 4.5 for 5 simulated copies. Both represent an increase in the absolute difference, but measure 90% of the simulated value). The high errors in Fig. 3 for *NBPF10*, *NBPF14*, *NBPF19,* and *NBPF20* are due to the high degree of sequence similarity between these genes. Reads belonging to these genes often map to one another as shown in Fig. 2. The grouping strategy employed here reduces the errors for *NBPF10*, *NBPF14*, *NBPF19,* and *NBPF20* as well as for *NBPF4*, *NBPF5P*, and *NBPF6*.

### Evaluation of alignment strategies

Using simulated data, we evaluated the accuracy of several alignments strategies, e.g. how well all DUF1220 copies in the genome are accounted for, and whether the aligned reads can be resolved into their respective clades. The alignment strategies we tested were; 1) ‘best alignment’ method (i.e. try to find the best possible alignment for each read, in the case of multiple valid alignments, one of the alignments is chosen at random), 2) ‘Align All’ method, (i.e. shorten longer reads to 36 bases, find all possible alignments within 2 mismatches, and normalize coverage at each location by dividing by the total number of domains measured); 3) ‘total counts’ strategy (as for 2) but using the Bowtie1 aligner; 4) multi-read correction method, (i.e. the contribution of each read is divided by the number of loci it aligned to. In this strategy, we first attempted to find the best possible alignment for each read using Bowtie1, but in the case of multiple valid alignments, all ties are kept rather than choosing one at random).

The data shown in Table 1 illustrate the accuracy of the copy number estimation based on simulated reads from all canonical DUF1220 domains aligned to the reference. The simulation was conducted ten times and the measured number of copies was compared with the known copies. This allows for the quantitative comparison of ‘off-target’ alignment rates at the level of domain, gene, group and clade shown in Table 2.

**Table 1:** The Root Mean Squared Error (RMSE) for the alignment strategies tested

Alignment Strategy	Total	CON1	CON2	CON3	HLS1	HLS2	HLS3
	(542)	(66)	(32)	(32)	(136)	(130)	(146)
Best Alignment	3.66	1.55	0.91	0.26	0.99	1.9	1.67
Multiread Correction	7.73	1.19	1.14	0.31	1.75	2.96	2.93
Align All/bowtie1	46.8	18.4	9.6	66.2	37.1	34.7	32.3
Align All/mrsFast	103.1	37.8	8.7	23.5	42.8	40.5	42.7

**Table 2:** Percent off-target alignments for the align all and best align strategies

	Clade	Domain	Gene	Group	Clade
Align All/mrsFast	CON1	88.1%	85.6%	75.0%	0%
	CON2	89.1	89.1	83.7	0
	CON3	94.7	94.7	91.7	49.9
	HLS1	98.8	88.5	45.7	46.8
	HLS2	98.8	87.7	42.8	38.9
	HLS3	98.7	85.4	48.1	25.1
Best Align	CON1	20.3	17.4	5.1	0
	CON2	24.8	24.8	16.0	0
	CON3	26.3	26.3	15.5	6.4
	HLS1	63.9	23.3	2.7	2.1
	HLS2	64.8	22.4	4.7	2.4
	HLS3	63.3	24.8	7.5	0.5

Overall the ‘best’ alignment strategy outperformed the other methods. For 566 total diploid DUF1220 copies, the ‘best’ alignment method was off by an average of 3.7 copies, the multiread correction method was off by 7.7, and the total alignment strategies were off by hundreds of copies. While the multiread correction method was competitive in terms of accuracy, it was the least computationally efficient. The ‘Align All’ method with mrsFast [26] performed better for CON2 than with other domains, which is likely due to the conservation of the sequence across genes. If *NBPF3*, *NBPF13P*, and *NBPF17P* are excluded, the error for CON2 estimate by ‘Align All’ drops to 0.08, which is lower than the ‘best’ alignment strategy.

**Fig. 5:**
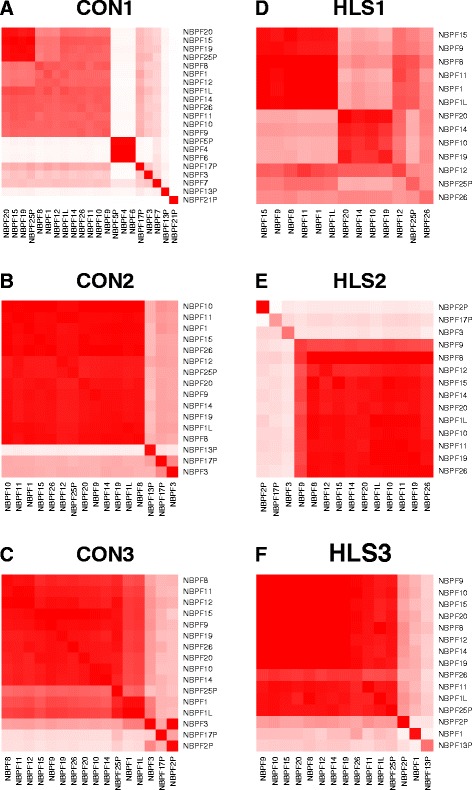
Read alignment ambiguity between *NBPF* genes using the ‘Align All’ strategy with mrsFast for the spike-in simulation study. For each clade (a-f), simulated reads from individual domains (*columns*) were mapped back to the reference genome and the percentage of reads aligning to each domain was calculated (*rows*). The *shade of red* is proportional to the percentage of reads aligning to each gene (*solid red* = 100% alignment and *white* = 0% alignment)

While the ‘Align All’ strategy was good at summarizing copies of CON1 and CON2 at the clade level, where 0% of the reads were found as off-target alignment, it lacked the ability to resolve copy number estimates below the clade level (e.g. if an extra CON2 domain was detected at the clade level, it would be difficult to tell from which gene it came) (Table 2). The ‘Align All’ strategy underestimated CON1 copy number, which is likely caused by reads not uniformly aligning to all domains (Fig. 5). For clades CON3, HLS1, HLS2 and HLS3, the ‘Align All’ strategy had a much higher off-target percentage than the ‘Best Align’ strategy (25–40% vs 0.5–7%) and the estimated copy numbers were higher than expected. This is likely due to the sequence similarity between CON3 and the HLS clades. Thus, it is likely that HLS domains inflated the measure of CON3 and of themselves. The ambiguity among HLS domains was much higher when using the Bowtie1 for the ‘Align All’ strategy. If we normalize the HLS clades by the total number of HLS1, HLS2, HLS3 copies, we can reduce the observed error to 46.8 overall DUF1220 copies. While the overall accuracy increases, it comes at the cost of distinguishing between HLS clades. Perhaps with an improved normalization scheme, the error in the copy number estimates from the ‘Align All’ strategies could be further reduced, however it is unlikely to be able to distinguish beyond the clade level.

### Validation by ddPCR

**Fig. 6:**
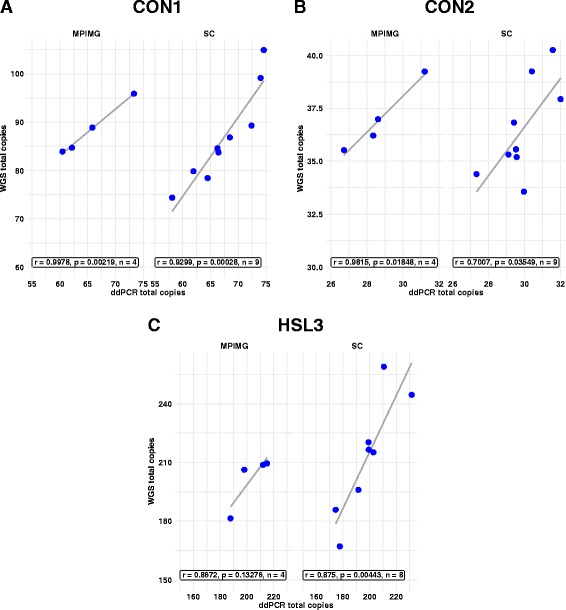
Correlation between whole genome sequencing and ddPCR copy number measurements for three representative DUF1220 clades; a CON1, b CON2, and c HLS3. Data shown is the ‘Best Align’ prediction of copy number for select samples from the CLM population (each point represents a single person) of the 1000 Genomes dataset, split by sequencing center, plotted against the measured copy number by ddPCR, using primers specific for each of the three DUF1220 clades. MPIMG = Max Plank Institute for Molecular Genetics, SC = Wellcome Trust Sanger Institute (SC). The Pearson correlation coefficient *r* is shown

The accuracy of our read depth method, and the ability to detect changes in copy number, were evaluated by comparing the read depth generated copy numbers of three DUF1220 clades (CON1, CON2 and HLS3) with the copy numbers measured by ddPCR (Fig. 6). We believe these three subtypes provide the best validation for the WGS estimates for the following reasons. Previously published ddPCR data for CON1 [17–19], CON2 [20], and HLS1 [17–19] suggested that measuring CON1, CON2, and one of the HLS clades would capture the range of copy numbers seen by all 6 DUF1220 clades. Any one of the HLS clades is likely to have a very similar copy number range as the others because, in hg38, they almost always occur as a triplet. HLS3 was chosen to represent the HLS clades for validation of our method because it has a lower off-target alignment rate than HLS1 and HLS2 (Table 2) Likewise, the off target alignments for CON1 and CON2 are zero at the clade level which make them ideal candidates for comparison. We have previously optimized the use of ddPCR for measurement of DUF1220 clade copy number and shown that the method is highly reproducible [17, 18, 20]. Because of batch effects between sequencing centers, we measured the Pearson correlation coefficients for each center separately.

As shown in Fig. 6, we observed high correlation coefficients for samples from the Max Plank Institute for Molecular Genetics (MPIMG) and the Wellcome Trust Sanger Institute (SC) for all three clades. The *p*-values were less than 0.05 in all but one test (Fig. 6). For samples from MPIMG, the *p*-value for the correlation between read depth and ddPCR for the HLS3 clade was 0.13. However, for the same samples, the *p*-value for the correlation between read depth and ddPCR for total HLS (HLS1, HLS2, and HLS3 combined) is 0.03. This improvement is likely due to the fact that combining the three HLS domains improves the accuracy of the read depth measurement, as there is a high degree of sequence similarity between HLS1, HLS2, and HLS3. Because there were only 4 samples in our analysis for MPIMG, the small change in read depth accuracy has a large effect on the *p*-value for these centers (but only a small effect on the *p*-value for samples from SC).

We observed that samples from the Baylor College of Medicine had a low correlation with ddPCR (Additional file 2: Figure S1). We hypothesize that this is due to the low mean insert size of these samples (226 bp whereas the others was between 450 and 500 bp). Due to this low correlation, we excluded these samples from further analysis in this study and recommend that samples from this sequencing center not be used in future read-depth based analysis of DUF1220 copy number. The agreement between ddPCR and read depth generated copy numbers for the remaining sequencing centers suggest that our methodology can reliably detect copy number changes of DUF1220 sequences.

### Application of read depth analysis to WGS data from the 1000 Genomes Project

**Fig. 7:**
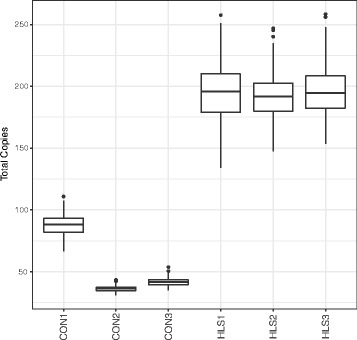
Genomic clade copy number for the six primary DUF1220 clades in 282 individuals from the 1000 Genomes project. Box plots of the total copies measured for each clade using the ‘Best Align’ strategy as described in the text, showing the median (*horizontal line*) and the upper and lower quartiles as the box with whiskers extending to 1.5 times the interquartile range with outliers shown as individual points outside this range. The variance shown include sequencing center bias and differences in populations. See Additional file 2:Figure S2 for results split by sequencing center and population

As a real-world application, we used the data from ~300 individuals from the 1000 Genomes Project to measure *NBPF* gene and DUF1220 clade copy numbers. Analysis of DUF1220 clade copy numbers shows the predicted distributions of CON1 and HLS1 DUF1220 clades based on prior studies (Fig. 7) [10]. The limited variation of CON2 and CON3 and the high variation of HLS domains is expected given the quantity of each clade in the reference and the relative copy number stability of each clade. The main sources of systematic variation from the sequencing center batch effects and the variation between populations are shown in Additional file 2: Figure S2.

**Fig. 8:**
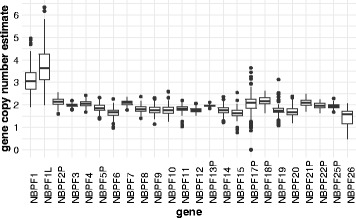
Estimation of copies of *NBPF* genes based on unique non-DUF1220 regions for 282 individuals from the 1000 Genomes project. Box plots of the total copies measured for each clade using the ‘Best Align’ strategy as described in the text, showing the median (*horizontal line*) and the upper and lower quartiles as the box with whiskers extending to 1.5 times the interquartile range with outliers shown as individual points outside this range. The variance shown include sequencing center bias and differences in populations

We also sought to measure DUF1220-containing gene copy number (as distinguished from intragenic DUF1220 domain copy number changes) by identifying sequences that were unique for each of the 30 DUF1220-containing genes, and outside of the DUF1220 domain regions. We simulated 100 bp paired-end reads from the entire human reference genome and aligned them back requiring a single unique match within one mismatch using Bowtie1, producing a set of unique regions within and around the coding portion of each *NBPF* gene (Additional file 3). To generate our estimates of gene copy number, we excluded any regions within the coding portion of the gene to avoid the possibility of intragenic changes affecting our gene-level estimate. We also excluded some untranslated exons (UTR) at the 5’ and 3’ ends of genes because our results indicated that these may reflect copy number changes of specific upstream or downstream regions that are not due to gene copy number changes. The average normalized read depth across these unique regions was calculated to estimate the gene copy number of each of these genes. Our gene copy number estimates are largely in agreement with those previously reported [21], including an elevated mean copy number and high variability for *NBPF1* (mean: 3.11 (95% CI: 3.04–3.18), min: 1.91, max: 4.96) (Fig. 8). Sudmant (2010) reported a copy number range for *NBPF1* of approximately 4 to 15 copies. We found that the sequence currently annotated as LOC102724250 on an un-placed contig, chr1_KI270711v1_random, is an *NBPF* gene with high similarity to *NBPF1*, which we refer to as *NBPF1L* in this paper. This may account for one of the missing copies of *NBPF1* previously described [21]. If the copy number reported for *NBPF1* in the Sudmant (2010) paper included both *NBPF1* and *NBPF1L*, then our equivalent range (*NBPF1* plus *NBPF1L*) is ~4 to ~11 copies which is fairly close to the range reported by Sudmant, 2010. By investigating the copy number of unique regions within and surrounding the coding portion of *NBPF1*, we found that some regions upstream of the coding sequence have copy numbers much greater than the regions closest to the coding sequence (Additional file 2: Figure S3), suggesting duplication of these regions independently of the protein-coding portion of the gene. We have excluded these regions from Fig. 8 where we report the gene copy numbers. This finding highlights the value of our in-depth analysis of *NBPF* and DUF1220.

### Comparison to previously published data

To further validate our read-depth method, we compared our copy number estimates with those published previously [21] for select multi-copy genes (*NSF*, *KIAA1267*) using the same 1000 Genomes Project samples as those in the comparison study. Values we obtained were highly-correlated with those previously reported (the Pearson correlation coefficient was 0.92 for NSF and 0.95 for KIAA1267). We also found that while our read depth-predicted distributions of *NBPF1* and *NBPF7* gene copy number among 1000 Genomes samples are similar to those previously reported [21], those we generated for *NBPF14* were not. To address this discrepancy, we assessed *NBPF14* copy number by ddPCR and found that our read depth and ddPCR estimates were very similar for most samples tested (Additional file 2: Figure S4), and were more concordant than the values previously reported. One explanation for the difference in *NBPF14* copy number between our study and Sudmant (2010) [21] may be the use of different genome assemblies [11]. The most recent assembly (hg38), which was used for our study, has a more completely finished 1q21 region [16] where the majority of *NBPF* genes reside. The hg38 assembly includes a better annotation of *NBPF14*, where *NBPF14* expanded from 2 HLS triplets (in hg19) to 7 triplets in hg38. *NBPF14* sequences were either missing from the genome reference or were improperly assigned when the previous estimates were reported by Sudmant et al. 2010 [21].

## Discussion

We describe a novel approach for the copy number measurement of DUF1220 protein domain family sequences and the genes that encode them (primarily *NBPF*) from WGS data. While some DUF1220 copies are indistinguishable from one another [10], the method described allows the 302 haploid copies of DUF1220 (hg38) to be accurately quantified at multiple levels of increased resolution (clade, group-specific clade, and in some cases, individual domains).

Through computational simulations, we were able to identify the sequencing and alignment parameters that lead to optimal measurement of DUF1220 copy number in *NBPF* genes. When we tested multiple alignment strategies for their alignment rates, accuracy and precision and observed different levels of performance for the different methodologies used. The alignment strategies tested had different strengths. For example, the ‘Align All’ strategy worked well to summarise the overall copies of CON1 and CON2 domains where zero off-target alignments were detected. However, errors were much higher for CON3 and HLS1–3 due to greater sequence similarity amongst domains from these clades. The off-target rates for these domains were between 25 and 50%. This effect can be partially mitigated by the ‘Best Align’ strategy. Among our key findings was the determination that longer sequence read-lengths increased our ability to reliably follow copy number changes for specific DUF1220 domains. This may seem obvious, but it differs from a commonly employed model that shortens reads to 36 bp [21, 24, 25]. We also determined that, while it is difficult to accurately measure most individual DUF1220 domains, we can accurately quantify the number of domains within each clade and within each gene (or small group of genes). The strategy of finding all possible alignments for each read as a summarization strategy was found to be inaccurate due to the heterogeneous nature of the sequence similarity of the domains. Finding the best possible alignment for each read and choosing among the ties at random was the most accurate and the most computationally efficient strategy. The ‘best’ alignment strategy along with the use of long sequencing reads allowed for the highest specificity and lowest off-target alignments.

To our knowledge, this is the first high-throughput method that allows DUF1220 copy number to be measured with clade-specific resolution, and the first method to measure clade copy number within specific *NBPF* genes. The ability to obtain clade-specific resolution has considerable significance in that variation in the copy number of *specific* DUF1220 clades has previously been shown to be associated with important phenotypic variation related to brain size [11–14, 27], disease [17–19] and cognitive function [20], and this method should allow future studies to be carried out with greatly increased speed and cost-effectiveness relative to previous methods (e.g. aCGH, ddPCR). For example, the large WGS datasets that are being generated for autism [28, 29] and other brain-related disorders should provide a rich resource to which the DUF1220 read depth approach can be applied. Applications of this method to study the biological variation of DUF1220 across the human population are currently underway and will be reported in follow on publications.

## Conclusions

The significance of the work presented here is in the development and validation of a computational, WGS strategy to estimate copies of DUF1220 domains, at the clade, gene, and domain level. As we demonstrate, previously published computational methods for measuring CNVs lack the ability to resolve DUF1220 into clades or other smaller groupings. The method we describe here is a great improvement to the published methods through improved alignment and summarization strategies. The ability to measure gene-specific clade groups allows researchers to test hypotheses related to the effects of DUF1220 changes in specific *NBPF* genes, which may reveal important disease associations not previously open to investigation. Because we can also determine gene copy number independently of DUF1220 domain number, this method allows the researcher to discriminate between CNVs involving gene duplication/deletion events and changes involving duplications/deletions of exons within a gene. Since DUF1220 domains show the greatest human lineage-specific copy number increase of any coding region of the genome, the strategies employed here and the insights we obtained should serve to guide other efforts to use read depth to measure copy number of highly duplicated sequences. The result of the work presented here is a greatly enhanced capability to analyze the role that these sequences play in human variation and disease. This method can find additional applications in high-resolution analysis of other multi-copy gene families and of genes containing multiple duplicated domains, though this was outside of the immediate scope of the work presented here.

## Methods

### Simulation studies

To assess the degree of read alignment ambiguity between DUF1220 domains, a ‘spike-in’ study was conducted, where reads from an individual domain were simulated and aligned back to the genome. Single and paired-reads, ranging in lengths from 36 bp to 300 bp, were randomly sampled from the reference genome (hg38). To simulate duplication or deletion events, the number of reads were varied to simulate one to ten copies of each DUF1220 domain. To obtain reads for a single domain, reads overlapping a DUF1220 domain of interest were isolated and aligned back to the genome using each of the alignment strategies below. Afterwards we compared the degree to which reads aligned to the expected location.

To assess the ability of each algorithm to account for all 271 haploid DUF1220 copies, a ‘baseline’ study was conducted where all canonical DUF1220 domains were simulated at diploid coverage and aligned back to the genome. Reads were simulated as described for the ‘spike-in’ study but with 100 bp paired-end reads. 100 bp paired-end reads were chosen because this is the sequencing length and type available from the 1000 Genomes Project.

For both simulation studies, the number of reads was adjusted to give a baseline diploid coverage of 30×. For paired-end reads the insert size was varied to match the variation found in the 1000 Genomes Project, normally distributed with a mean insert size of 350 bp and a standard deviation of 50 bp.

Sequencing errors and quality scores could potentially increase the ambiguity of each read and impact the ability to distinguish between DUF1220 domains. Quality scores from Illumina sequence data tend to decrease towards the end of each read. To model this, we measured the mean quality score at each base for the 1000 Genomes fastq files and used loess regression to model the distribution. The profile was extended so that each simulated read length would have the same quality score profile. This was done to simulate the quality score drop off rate relative to read length observed in data obtained from different generations of Illumina sequencers (GAIIx, HiSeq2000, MiSeq, etc). Each sequencing pair was modeled separately, since the second read tends to have lower quality scores than the first. Sequencing errors were modeled as described in [30]. The mean probability for a sequencing error for the first read was 0.0026 and 0.004 for the second pair. The error rate was increased linearly such that the probability of a sequencing error was 1.5 times more likely at the end of the read and 1/2 as likely at the beginning of the read.

For alignment to the human genome reference, we tested various alignment strategies. Bowtie2 (version 2.2.9) [31] was used to find the ‘best’ alignment for each read, with the ‘--very-sensitive’ preset and a max-insert size of 800 bp. For the ‘All Align’ strategy, mrsFast-Ultra (version 3.3.11) [24] was used as described in [25], with the parameters ‘--crop 36’ and ‘-e 2’ to crop 100 bp reads to 36 bp and aligned with up to two mismatches. As an alternative, Bowtie (version 1.1.2) [32] was also used for the ‘All Align’ strategy, with the following parameters ‘--all -v 2 -X 800’. For the multiread strategy, reads were aligned to the genome using Bowtie v1.1.2 with the ‘--best --strata --all --v 2’ parameters. In this case, Bowtie attempts to find the best possible alignment for each read. If multiple valid alignments are found, rather than choosing one at random, all ties are returned. Later the contribution of each read can be weighted as described below.

After alignment, the BAM files were converted to BED format using bedtools (v2.17.0) [33]. Paired-end reads aligned as a proper pair were joined into a single fragment and discordant pairs were treated as single-end reads. The lengths of the discordant reads were extended to half mean insert size for that sample following a normal distribution. The resulting fragments were then intersected with each DUF1220 domain using bedtools. For the multi-read correction, the resulting BED file was sorted by read name and the number of bases overlapping with each DUF1200 domain was divided by the number of places each read aligned. The coverage for each DUF1220 domain was calculated by dividing the number of bases overlapping the domain by the domain length. The number of copies of each domain was calculated by dividing the coverage by the expected simulated haploid coverage, in this case 15× coverage. For the ‘Align All’ strategy, the copy number was further normalized by the total number of domains for each clade. The Root Mean Squared Error was calculated by the following formula: RMSE = sqrt(sum((measured.copies – expected.copies)^2))

### Analysis of sequence data from the 1000 genomes project

**Fig. 9:**
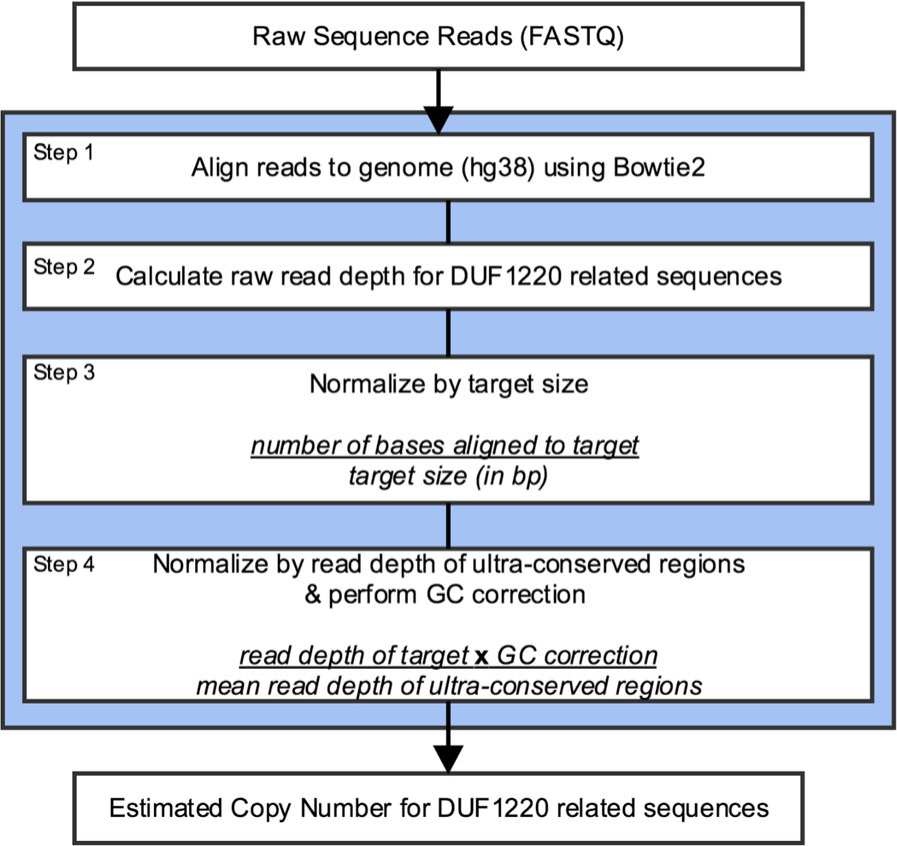
Data processing steps for estimation of DUF1220 related sequences

Raw sequence data were obtained from the 1000 Genomes Project [34] via ftp download from EBI ftp://ftp.sra.ebi.ac.uk/vol1/fastq. The full list of sequence data was obtained from ftp://ftp.1000genomes.ebi.ac.uk/vol1/ftp/data_collections/. Approximately 25 individuals were randomly chosen from each of the CEU, YRI, CHB, JPT, MXL, CLM, PUR, ASW, LWK, CHS, TSI, IBS, FIN, and GBR populations for a total of 324 individuals. Individuals from the CEU, YRI, CHB, and JPT populations were selected to match with those reported previously [21]. The data were obtained from the Illumina 2000 with 100 bp paired-end reads, with an average of 139 million reads and 15× coverage per genome. The reads were filtered and trimmed to remove low quality bases (Phred score < 10) from the 3′ ends of the read using Cutadapt (version 1.31) [35] (cutadapt –a XXX –A XXX –q 10 --minimum-length 80 --trim-n). Reads trimmed shorter than 80 bases were removed (on average 18.5 million reads per sample). Samples with less than 10× coverage were removed from the analysis. Coverage was calculated by multiplying the number of filtered reads by the insert size and dividing by the number of bases in the human genome reference. The sequence data were analyzed following the ‘best’ align strategy as described above and outlined in Fig. 9. For copy number estimation, the genomic coordinates spanning the short and long exons of each DUF1220 domain were combined. Where the domains were more than 1 kb apart, the boundaries of the domains were extended up to 250 bp to allow the possibility of capturing unique sequence directly adjacent to the domain. Sequence coverage for each region of interest is then normalized by dividing the coverage for every region of interest by the mean coverage of highly conserved regions and multiplying the normalized value by a GC correction factor. To derive GC correction factors, the genome including highly-conserved regions were binned into 1 kb windows and the read depth is plotted against the %GC content. A Loess regression model is fitted to the data to determine the correction factor for each GC bin. The background regions used for normalization and GC correction were derived by merging regions from our simulations that map uniquely to the human genome reference within two mismatches along with regions from the database of Ultra-Conserved Elements (UCE) [36]. Any regions found in the Database of Genomic Variants [37] were subtracted from the background regions.

### Annotation of DUF1220 domains and DUF1220 containing genes in hg38

#### Identification of DUF1220 domains

The genomic coordinates of DUF1220 domains in the reference genome are required to carry out WGS read depth. To identify DUF1220 domains and their genomic coordinates, we utilized HMMER version 3.1b2 [38] and components of a pipeline published by Zimmer & Montgomery (2015) [11]. Briefly, HMMER was used to generate a hidden markov model based on the DUF1220 (PF06758) seed domains present in the pfam database as of July 10th, 2017 (Additional file 4) [39]. This hidden markov model was then used to search the longest isoforms of all proteins in the human proteome (Ensemble v.81) [40] for matching domains with an expectation value (E-value) less than 1e^−10^. The cDNA sequences corresponding to the protein domain hits were then aligned with MAFFT and this alignment was used to generate a nucleotide hidden markov model. The nucleotide hidden markov model was then used to search the reference genome (hg38) for DUF1220 domains with an expectation value (E-value) less than 1e^−10^, producing a list of genomic coordinates for DUF1220 domains in the reference genome. Custom scripts were used to convert the HMMER output files to bed files, appropriately account for both exons in a DUF1220 domain, and assign DUF1220 domains to the appropriate clade.

#### Locations of DUF1220 domains in hg38

Consistent with the results presented by Zimmer and Montgomery (2015), we identified 302 DUF1220 domains in hg38 using HMMER. A complete list of DUF1220 domain coordinates can be found in Additional file 5. Refseq (GCF_000001405.35_GRCh38.p9_genomic.gff) and Ensembl (Homo_sapiens.GRCh38.86.gtf) exon annotations differ slightly for the DUF1220 containing genes. For each gene, the reference annotation that most completely matches the known structure of DUF1220 domains was used. If both references were identical with respect to DUF1220 exon annotation, the reference with the greater number of UTR exons was utilized.

DUF1220 domains are known to be composed of an exon doublet consisting of a short and long exon of characteristic length. We observed that the coordinates returned by HMMER overlapped only single exons (the long exon). This is likely because the search algorithm is unable to identify the N-terminal end of the domain (encoded by the small exon) across the intronic gap. We confirmed that in almost every case, the short (50 – 111 bp) exon immediately preceding the exon identified by the HMMER coordinates codes for the N-terminus of the DUF1220 domains. Our custom scripts annotate each appropriate exon pair as belonging to the same DUF1220 domain.

#### Assignment of DUF1220 domains to appropriate clade

As previously described, the majority of DUF1220 domains can be divided into 6 clades [10]. The domains of each clade can be distinguished by their position within the gene, their exon lengths (Additional file 5), and protein sequence motifs unique to each clade (Additional file 6). We assigned each DUF1220 domain to a clade based on the presence or absence of these characteristic protein sequence motifs. The validity of our clade assignments can be confirmed by viewing a phylogenetic tree of the protein sequences (Fig. 1). Furthermore, because the amino-acid motifs particular to each clade are highly conserved within clades, it is easy to view the distinctions between clades by viewing the aligned protein sequences (Additional file 6).

Some (16/302) DUF1220 domains do not fit well within the previously established clades, but clearly form 5 distinct clusters based on sequence similarity. We have therefore established five new clades referred to as CON4–8 (Additional file 5). In contrast to the domains belonging to the six clades described above, the majority of these DUF1220 domains are located between 1p11.2 and 1p13.3. These were not analyzed in this study because of their non-canonical nature and their locations predominately within non-*NBPF* genes. A few (6/302) DUF1220 domains appear to be hybrid domains, that is, they contain a short exon characteristic of one domain and the long exon characteristic of a different domain. These domains were not included in our analysis.

Individual DUF1220 domains are referred to by their gene name, the name of the clade to which the domain belongs, and a number reflecting the placement of that domain within the gene. For example, NBPF1_CON1_3 refers to the third CON1 domain within *NBPF1* and NBPF20_HLS1_8 refers to the eighth HLS1 domain within *NBPF20*. Six DUF1220 containing genes currently lack formal gene names in either RefSeq or Ensembl but each of these has high sequence similarity to another gene (e.g. LOC102724250 is very similar to *NBPF1*). For clarity, in Additional file 5 and Additional file 2: Figure S1, we refer to these genes by descriptive names reflecting their similarity to named genes (LOC102724250: *NBPF1L,* LOC100996724: *PDE4DIPL1*, RP11-744H18.1: *PDE4DIPL2*). The three genes containing CON8 domains are similar to one another but not to any currently named gene, so they are referred to as CON8 containing 1, 2, and 3 (LOC105369199: *CON8C1,* LOC105369140: *CON8C2*, LINC00869: *CON8C3*). In Additional file 5 we also label some exons as conserved exon 1–7 (CE1-CE7) because the sequence of these exons is highly conserved across genes and, for CE1-CE3, at multiple locations within genes. Several non-coding exons also have high sequence similarity across genes and these are labeled UTR1-UTR20 (e.g. the sequence of UTR13 exons is highly conserved across different genes). Exons that do not meet any of the conditions described above are referred to as “exon” with a number denoting the exon position in the gene.

### Measurement of DUF1220 by digital droplet PCR (ddPCR)

We performed ddPCR essentially as previously described [17] to validate our copy number estimates for three representative DUF1220 clades. DNA samples were obtained from Coriell Biorepository and digested with the restriction enzyme DDE1. Digested DNA, primers, and fluorescently labeled probes were then combined following the manufacturer’s protocol. Primer and probe sequences were as follows: CON1: Forward 5′ - AATGTGCCATCACTTGTTCAAATAG - 3′, Reverse 5′ - GACTTTGTCTTCCTCAAATGTGATTTT – 3′, Probe– 5′ – CATGGCCCTTATGACTCCAACCAGCC – 3′; CON2: Forward 5′ – ACCAATCTGCAGGAGTCTGA’ – 3′, Reverse 5′ - TACGAGGCCAACATTTCAGG – 3′, Probe 5′ – AGAGGAGGAAGTCCCCCAG -3′; HLS3: Forward 5′ - GAGGTAGTAGAGCCTGAAG – 3′, Reverse 5′ – CCCACGTCAAGAGAAAAGC – 3′, Probe 5′ - CCTGACTCCTGCCAGCCCTA - 3′; *NBPF*14: Forward: 5′ - AGAGTCCTGGGTGACATG – 3′; Reverse: 5′ – CCTGCTCCTCTCTATTCC – 3′; Probe: 5′ - CTCCTGACTCCTGACCTCTACA- 3′; RPP30: Forward – ‘GATTTGGACCTGCGAGCG’, Reverse – ‘GCGGCTGTCTCCACAAGT’, Probe – ‘TTCTGACCTGAAGGCTCTGCGC’. ddPCR cycle conditions are as follows: 95 °C – 10 min, 40× (94 °C – 30 s, annealing temperature (described below) – 60 s), 98 °C – 10 min, 12 °C – hold. ddPCR cycle conditions for different target sequences varied only in the annealing temperature utilized. For CON1 and HLS3, the annealing temperature was 56 °C, for CON2 the temperature was 61 °C, and for *NBPF14*, the temperature was 59.7 °C.

Within a ddPCR run, each sample was run in triplicate and the counts of positive and negative droplets from each of the replicate wells were combined before calculating the copy number for each sample. Each sample was run in this manner 3 (in some cases 4) times, and the mean copy number of these runs was utilized to calculate the correlation coefficient with WGS read depth.

Initial analysis included 44 samples from the 1000 Genomes Project analyzed for both CON1 and CON2 copy number. Because we identified that the short insert size of BCM samples affected the read depth accuracy, we did not analyze the BCM samples by ddPCR for HLS3. Two additional samples were excluded from the final analysis, and from ddPCR of HLS3, because their whole genome sequencing data was derived from a large number of lanes (HG01454 and HG01139 were sequenced across 35 and 48 lanes respectively) and a third was excluded because it had a lower than normal number of reads (HG01148). The final analysis included 13 samples for each of the DUF1220 clades.

### Source code

The source code used to analyze the 1000 Genomes data is available online at https://github.com/dpastling/plethora. And the source code used to carry out the simulations is available at https://github.com/dpastling/DUF1220_simulation. The source code used to annotate DUF1220 domains is available at https://github.com/IleaHeft/DUF1220annotator.

All source code used in this study is released under the MIT License and archived on Zenodo at http://doi.org/10.5281/zenodo.840606.

## Additional files

Additional file 1: Supplementary **Table S1**. (PDF 98 kb)

Additional file 2: Supplementary **Figures S1, S2, S3, and S4**. (PDF 485 kb)

Additional file 3: Unique non-DUF1220 regions for quantifying gene copies. (BED 25 kb)

Additional file 4: PF06758 seed domains. (TXT 7 kb)

Additional file 5: Annotation of DUF1220 domains and exons in hg38. (BED 49 kb)

Additional file 6: Alignment of DUF1220 proteins. (ALN 94 kb)
