# Supplementary material for: High resolution measurement of DUF1220 domain copy number from whole genome sequence data
Source: BMC Genomics. 2017 Aug 14;18:614. doi: 10.1186/s12864-017-3976-z (PMC5556342; doi:10.1186/s12864-017-3976-z)
Supplement: Supplementary file 1 — Supplementary Table S1. (PDF 98 kb) [file 12864_2017_3976_MOESM1_ESM.pdf]

Table S1. Gene specific clade groups (n = 60)

CON1 (n = 14)	CON2 (n = 11)	CON3 (n = 11)	HLS1 (n = 7)	HLS2 (n = 9)	HLS3 (n = 8)
Single Gene in Group					
NBPF3	NBPF3	NBPF2P	NBPF9	NBPF1L	NBPF2P
NBPF7	NBPF9	NBPF3	NBPF15	NBPF2P	NBPF9
NBPF9	NBPF15	NBPF9	NBPF25P	NBPF3	NBPF13P
NBPF13P	NBPF13P	NBPF15		NBPF9	NBPF25P
NBPF15	NBPF17P	NBPF17P		NBPF15	
NBPF17P	NBPF20	NBPF20		NBPF17P	
NBPF20	NBPF25P	NBPF25P			
NBPF21P					
NBPF25P					
Multiple Genes in Group					
NBPF4, NBPF5P, NBPF6					
NBPFF10, NBPF14, NBPF19	NBPFF10, NBPF14, NBPF19	NBPFF10, NBPF14, NBPF19	NBPFF10, NBPF14, NBPF19, NBPF20	NBPFF10, NBPF14, NBPF19, NBPF20	NBPFF10, NBPF14, NBPF19, NBPF20, NBPF15
NBPF1, NBPF1L	NBPF1, NBPF1L	NBPF1, NBPF1L	NBPF1, NBPF1L		NBPF1, NBPF1L
NBPF8, NBPF26	NBPF8, NBPF26	NBPF8, NBPF26	NBPF8, NBPF26	NBPF8, NBPF26	NBPF8, NBPF26
NBPF11, NBPF12	NBPF11, NBPF12	NBPF11, NBPF12	NBPF11, NBPF12	NBPF11, NBPF12	NBPF11, NBPF12
