# Supplementary material for: High resolution measurement of DUF1220 domain copy number from whole genome sequence data
Source: BMC Genomics. 2017 Aug 14;18:614. doi: 10.1186/s12864-017-3976-z (PMC5556342; doi:10.1186/s12864-017-3976-z)
Supplement: Supplementary file 2 — Supplementary Figures S1, S2, S3, and S4. (PDF 485 kb) [file 12864_2017_3976_MOESM2_ESM.pdf]

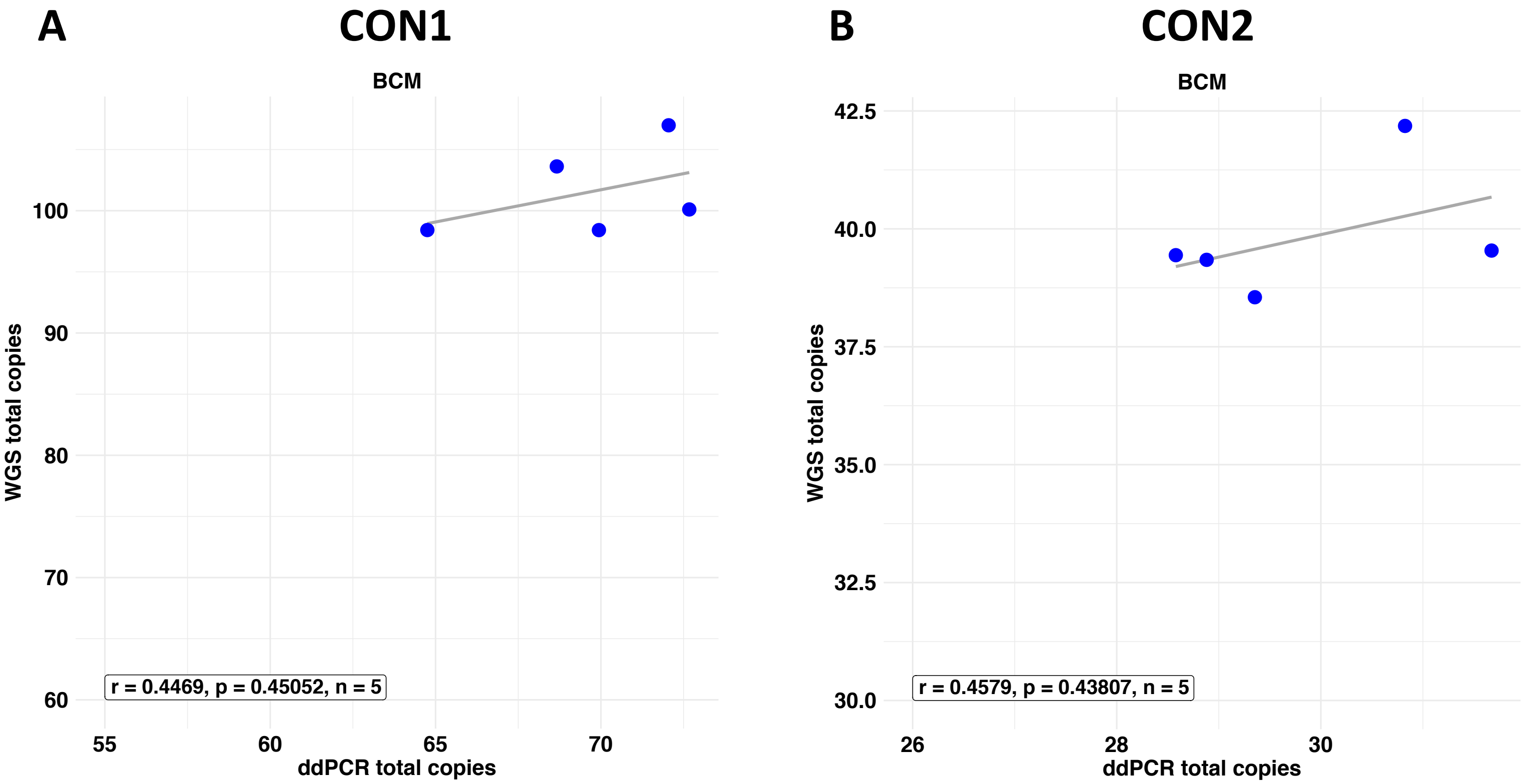

Figure S1: Correlation between whole genome sequencing and ddPCR for samples from the Baylor College of Medicine (BCM) for the clades A) CON1 and B) CON2. These samples were removed from the analysis described in Figure 7 because of the unusually short insert sizes for these samples. The Pearson correlation coefficients, p values, and sample sizes are shown on each plot. The axes are drawn to be consistent with Figure 7.

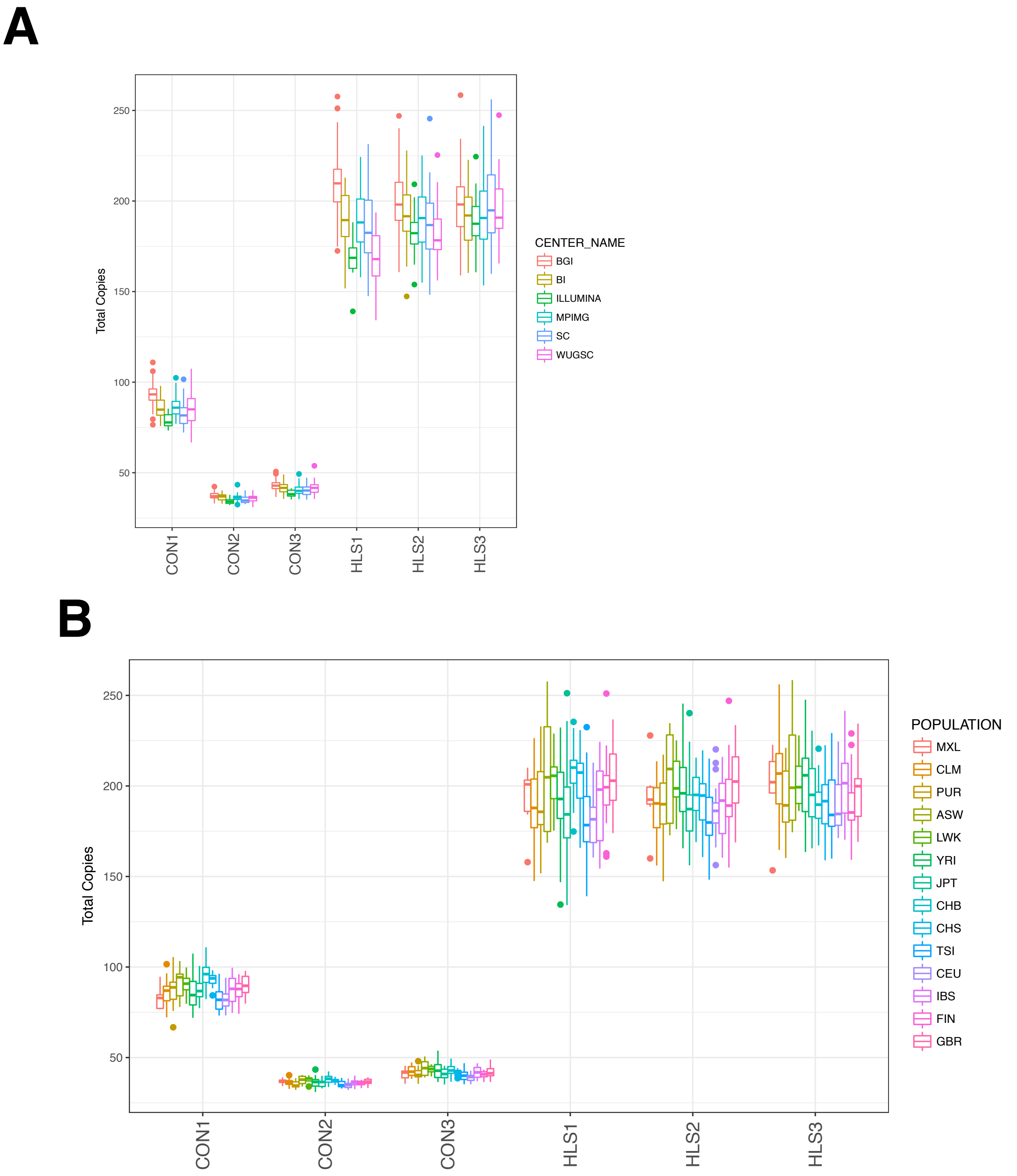

Figure S2: Main sources of variation in 1000 Genomes WGS data. A) Variation in copy number across sequencing centers. B) variation in copy number across populations. The data are the same as described in Figure 8, but have been divided into the respective categories.

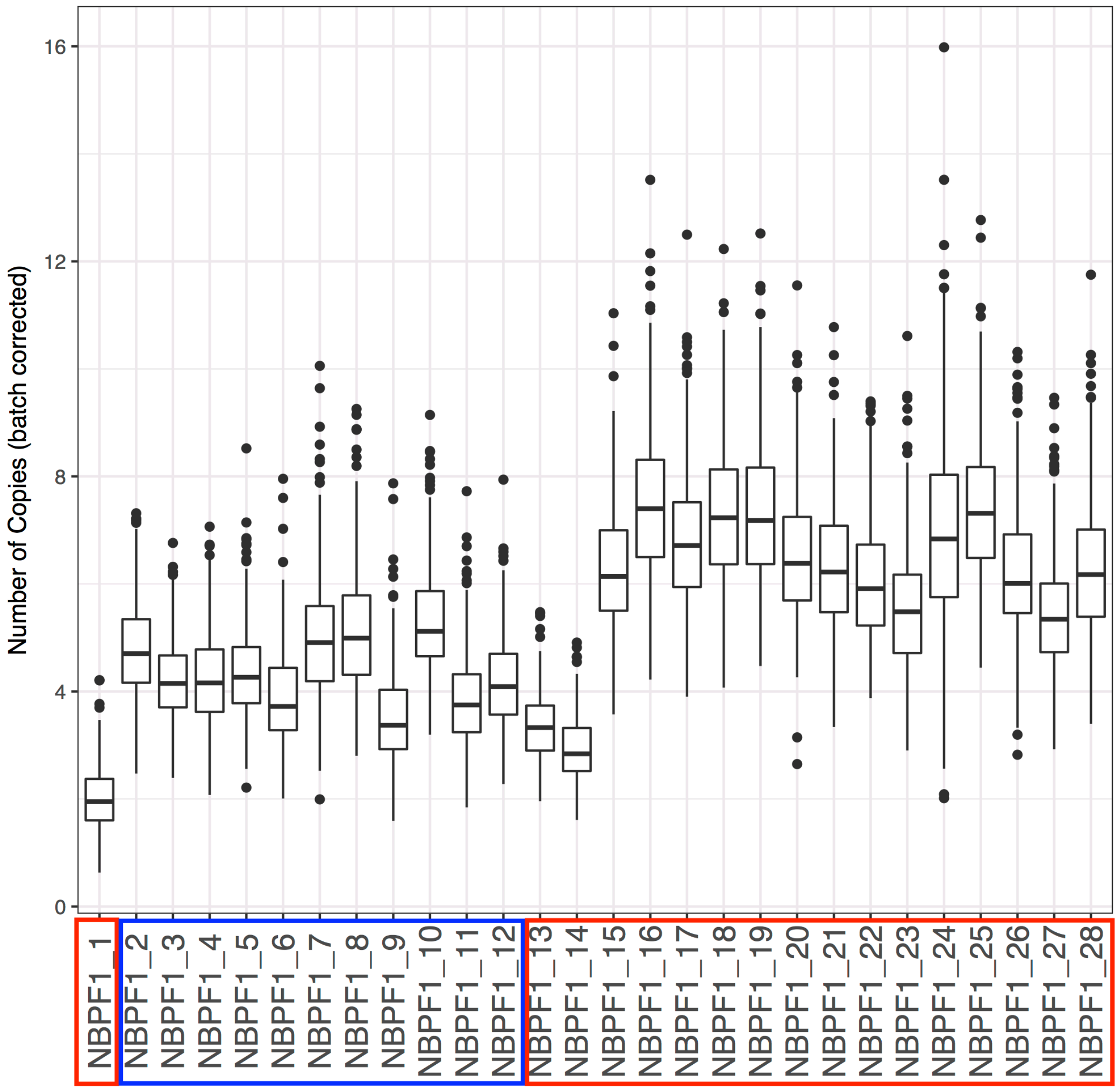

Figure S3: Copy number estimates for unique non-DUF1220 sequences around *NBPF1* from the 1000 Genomes data. Regions located within the boundaries of the gene are highlighted in blue, and regions located either upstream (regions 13 through 28) or downstream of the gene (region 1) are highlighted in red. The upstream regions 15 though 28 have an mean diploid copy number of 7, the internal regions 2 through 12 have a mean diploid copy number around 4, and the downstream region 1 has a mean diploid copy number of 2.

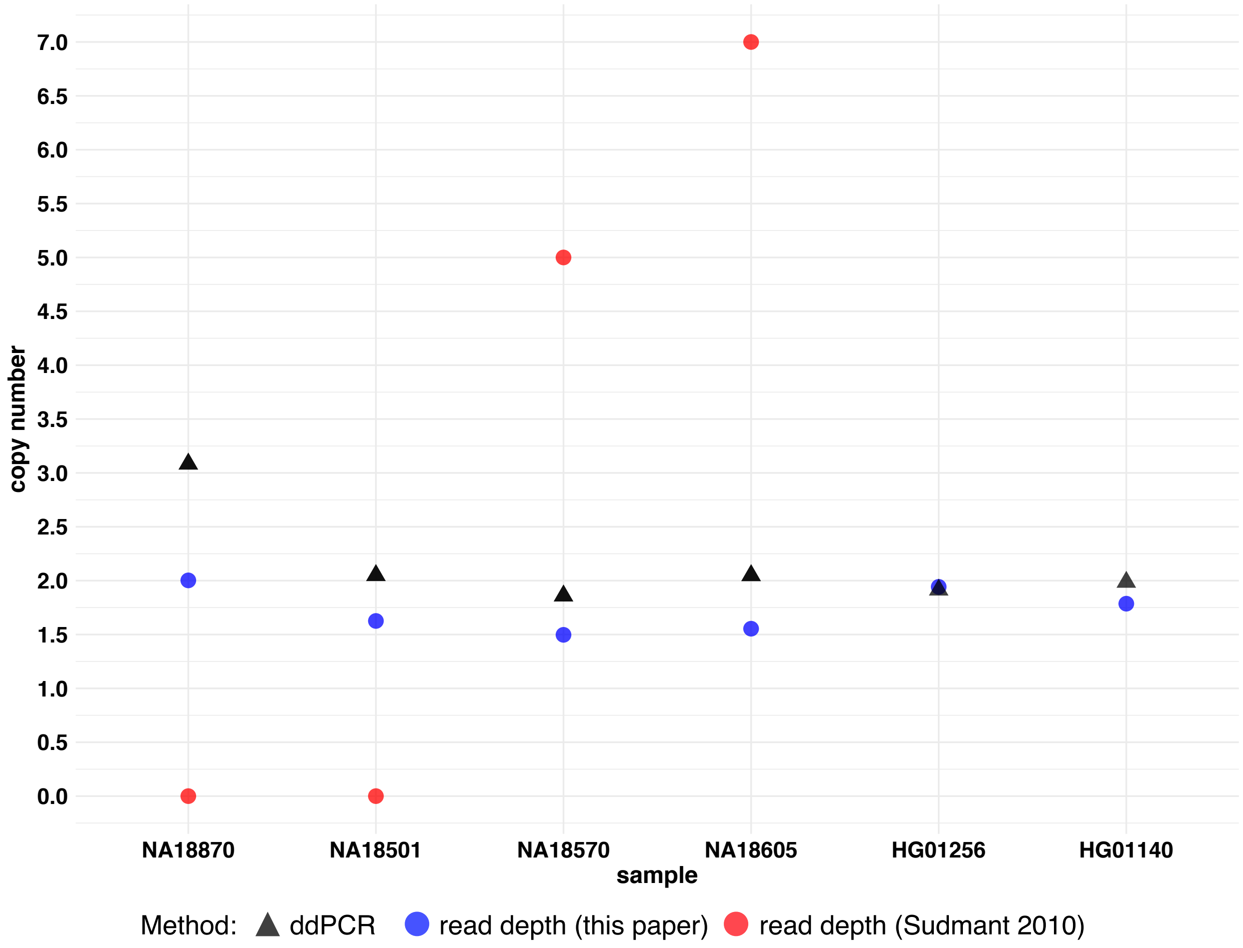

Figure S4: Comparison of NBPF14 gene copy number estimates by ddPCR (black triangles), the WGS method as described in this paper (blue circles), and the values reported in Sudmant, 2010 (red circles). Comparison between the method in this paper and ddPCR was conducted on six samples, four of which are reported in Sudmant, 2010. The two on the far right were not reported in Sudmant, 2010 but are included here for additional confirmation.
